# Fibrin with Laminin-Nidogen Reduces Fibrosis and Improves Soft Palate Regeneration Following Palatal Injury

**DOI:** 10.3390/biom11101547

**Published:** 2021-10-19

**Authors:** Doris H. Rosero Salazar, René E. M. van Rheden, Manon van Hulzen, Paola L. Carvajal Monroy, Frank A. D. T. G. Wagener, Johannes W. Von den Hoff

**Affiliations:** 1 Department of Dentistry, Orthodontics and Craniofacial Biology, Radboud Institute for Molecular Life Sciences, Radboud University Medical Centre, 6525EX Nijmegen, The Netherlands; doris.roserosalazar@radboudumc.nl (D.H.R.S.); Rene.vanRheden@radboudumc.nl (R.E.M.v.R.); Frank.Wagener@radboudumc.nl (F.A.D.T.G.W.); 2 Department of Medical Basic Sciences, Faculty of Health, Universidad Icesi, Cali 760008, Colombia; 3 Central Facility for Research with Laboratory Animals (CDL), Radboud University Medical Centre, 6525EZ Nijmegen, The Netherlands; Manon.vanHulzen@radboudumc.nl; 4 Department of Oral and Maxillofacial Surgery, Special Dental Care and Orthodontics, Erasmus Medical Center, 3015GD Rotterdam, The Netherlands; p.carvajalmonroy@erasmusmc.nl

**Keywords:** fibrin, soft palate, cleft palate, skeletal muscle, nidogen, regenerative medicine

## Abstract

This study aimed to analyze the effects of fibrin constructs enhanced with laminin-nidogen, implanted in the wounded rat soft palate. Fibrin constructs with and without laminin-nidogen were implanted in 1 mm excisional wounds in the soft palate of 9-week-old rats and compared with the wounded soft palate without implantation. Collagen deposition and myofiber formation were analyzed at days 3, 7, 28 and 56 after wounding by histochemistry. In addition, immune staining was performed for a-smooth muscle actin (a-SMA), myosin heavy chain (MyHC) and paired homeobox protein 7 (Pax7). At day 56, collagen areas were smaller in both implant groups (31.25 ± 7.73% fibrin only and 21.11 ± 6.06% fibrin with laminin-nidogen)) compared to the empty wounds (38.25 ± 8.89%, *p* < 0.05). Moreover, the collagen area in the fibrin with laminin-nidogen group was smaller than in the fibrin only group (*p* ˂ 0.05). The areas of myofiber formation in the fibrin only group (31.77 ± 10.81%) and fibrin with laminin-nidogen group (43.13 ± 10.39%) were larger than in the empty wounds (28.10 ± 11.68%, *p* ˂ 0.05). Fibrin-based constructs with laminin-nidogen reduce fibrosis and improve muscle regeneration in the wounded soft palate. This is a promising strategy to enhance cleft soft palate repair and other severe muscle injuries.

## 1. Introduction

Cleft lip and/or palate (CLP) is the most common congenital orofacial malformation with a worldwide incidence of 1:700 to 1:1000 live births each year [1,2]. Around 46% of all CLP cases have a cleft in the soft palate, which results in diminished functions such as swallowing and speaking [3,4,5]. Despite surgical repair, about 30% of all cases with a cleft in the soft palate experience persistent problems with speech [6]. Craniofacial muscles seem to regenerate less than other skeletal muscles and show extensive fibrosis [7]. This is mainly caused by transforming growth factor-β1 (TGFβ1) that induces the production of large amounts of collagen type I [3,8]. TGFβ1 also inhibits the development of new muscle fibers, by reducing the proliferation, differentiation and fusion of satellite cells (SCs), the stem cells of muscle tissue [3,8,9]. 

SCs are located between the sarcolemma and the basement membrane of the muscle fiber, and are surrounded by extracellular matrix (ECM) components including collagen type IV, glycoproteins and glycosaminoglycans [9,10,11]. Laminin and nidogen, formerly known as entactin, are crucial components of the basement membrane in skeletal muscles due to their binding sites for growth factors [12]. The laminins LN-111 and LN-211 contribute to the assembly of the basement membrane, cell signaling and force transmission during muscle contraction [13]. These components are produced by local fibroblasts in the healthy tissue, but also by SC-derived myoblasts during muscle repair [9]. In vitro, laminin-nidogen promotes myofiber formation on collagen coatings, indicating it may also be suitable to enhance muscle constructs [14]. 

Fibrin is a viscoelastic biomaterial with a variable stiffness that mostly depends on the concentrations of fibrinogen and thrombin [15]. Platelet rich fibrin (PRF) is an autologus substrate prepared from blood that contains multiple biomolecules including TGFβ1 [16]. The exact composition of PRF is therefore highly variable [17]. For this reason, we chose pure fibrin, which is an “off the shelf” product. Preclinical research using fibrin-based hydrogels in musculoskeletal defects and heart regeneration has shown promising results [18,19,20,21]. Bone defects in rabbits treated with fibrin showed extensive osteoblast proliferation and angiogenesis [21,22]. In vitro, bone marrow stem cells in fibrin hydrogels showed an elongated shape and spindle-like conformation during differentiation [21,23]. Fibrin has a high porosity and biocompatibility, and a low density that allows tissue ingrowth [21].

In skeletal muscle, fibrin has been used to deliver cells, ECM components, and drugs into extensive muscle injuries [20,24]. These in vivo studies show fibrosis reduction and increased myofiber formation [20,24]. Recently, fibrin enhanced with laminin-111 in a full-thickness excisional injury in a rat limb muscle showed improved muscle regeneration and fibrosis reduction 56 days after implantation [25]. Additionally, fibrin-based heart valves, cardiac patches, and grafts to deliver stem cells into injured cardiac muscle showed increased vascularization and cardiac function [18,19]. Fibrin-based constructs have not been used in the injured soft palate up to now.

In in situ tissue engineering, an acellular construct is implanted containing cues to attract local cells and guide their differentiation into the desired cell type [26]. This in situ strategy is a practical and low-cost method as no cultured cells are required. In the present study, we used a recently developed rat model for soft palate muscle regeneration and fibrosis that involves a 1 mm biopsy wound in the soft palate [27]. This model shows extensive fibrosis and impaired muscle regeneration up to 56 days after wounding, while EMG analysis also indicated impaired muscle function [28,29]. We implanted fibrin constructs with and without laminin-nidogen into the biopsy wounds. The aim of this study is to analyze the effects of these fibrin constructs on fibrosis and regeneration of soft palate muscles in rats. 

## 2. Materials and Methods

### 2.1. Animals

A total of 104 male wistar rats, 9 weeks old and weighing 250–320 g, were randomized and used for the experiments (8 rats per group as determined by a power analysis). The animals were housed two per cage. All rats received food and water ad libitum and a dark/light cycle of 12 h each. After surgical wounding, the animals received powdered chow in water during three days in addition to the standard food. The animals were divided into four groups. Group 1 (W) received an experimental 1 mm excisional wound in the soft palate without implant (n = 32). Group 2 (WF) received an excisional wound and implantation of fibrin alone (n = 32). Group 3 (WFL) received an excisional wound and implantation of a construct of fibrin with laminin-nidogen (n = 32). Group 4 (C) was an age control at 56 days (n = 8) without an excisional wound. The rats were marked by ear punching. Eight rats of each experimental group were euthanized at days 3, 7, 28 and 56 by the standard CO_2_/O_2_ protocol. The soft palates were dissected for histological analysis.

### 2.2. Fibrin Constructs

**Figure 1:**
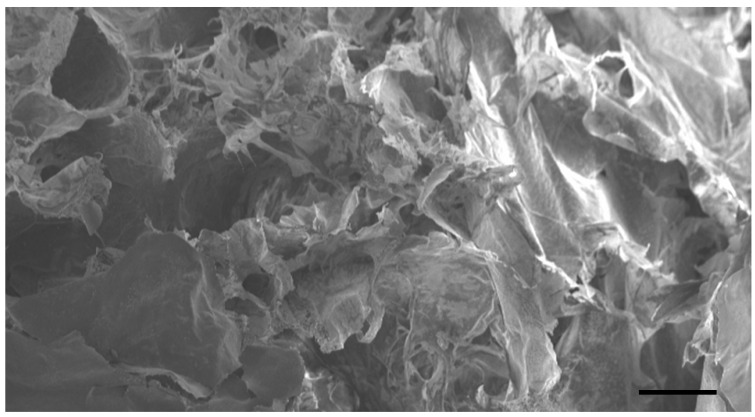
Fibrin construct. Fibrin gel (1 mL) was freeze-dried (Virtis SP Scientific BenchTop Pro, Warminster, PA, USA) for 48 h at 264 µBar at −50 °C, the sample was then prepared for SEM with a chromium coating of 10nm thickness. SEM was performed with Zeiss Gemini equipment at 5 kV. The scale bar is 200 µm.

Fibrin constructs were prepared by mixing fibrinogen (F8630, Sigma-Aldrich, St. Louis, MO, USA), thrombin (T7513, Sigma-Aldrich, St. Louis, MO, USA), aprotinin (A1153, Sigma-Aldrich, St. Louis, MO, USA) and calcium chloride (CaCl_2_) under sterile conditions. Fibrinogen was reconstituted in 0.9% NaCl at 37 °C and filtered through a 0.2 μm filter. Thrombin was reconstituted in BSA 0.1% and mixed with CaCl_2_ 4 mM (1:1). Aprotinin (Sigma-Aldrich, St. Louis, MO, USA) was diluted in PBS and mixed with the fibrinogen solution (1:1). The fibrin construct had final concentrations of 5mg/mL fibrinogen, 100 μg/mL aprotinin, 2.5 U/mL thrombin and 1 mM CaCl_2_ in PBS buffer. Figure 1 shows a scanning electron microscope image of a fibrin construct. Previous publications have shown that the stiffness of fibrin with 5 mg/mL and 2.5 U/mL thrombin is about 0.3 kPa (Structural Modulus) [30,31].

To prepare fibrin plus ECM components, laminin-nidogen (354259-Corning, Sigma-Aldrich, St. Louis, MO, USA) was added to an adjusted fibrin mixture to a final concentration of 100 µg/mL. The laminin-nidogen complex contains the laminins LN111 and LN211 also present in the basal lamina of the skeletal muscle tissue [13]. The concentration of 100 µg/mL we used previously showed improved myotube formation on collagen coatings in vitro [14]. A similar concentration of laminin showed improved myofiber formation in vivo in rats [25].

### 2.3. Surgical Wounding and Implantation

Presurgical and perioperative analgesia was provided as described [29]. In short, presurgical analgesia was provided in the drinking water 24 h prior to surgery containing 0.4 mL of carprofen (Rymadil, Zoetis, Parsippany, NJ, USA) in 300 mL of water. Perioperative analgesia was administered by a subcutaneous injection of carprofen (Rimadyl) 5 mg/kg. For anesthesia, a mixture of ketamine (Ketamine 10%, Alfasan, Woerden, the Netherlands) 37.5 mg/kg and atropine (Atropine sulfate, Pharmachemie B.V, Harlem, the Netherlands) 0.25 mg/kg diluted in saline 0.25 mL, was injected subcutaneously. Separately, non-diluted dexdomitor (Dexdomitor, Zoetis, Parsippany, NJ, USA) 0.25 mg/kg was injected subcutaneously, 25% oxygen in air was provided and body temperature and oxygen saturation were monitored. 

The excisional wound was made as previously described [27]. In brief, the surgical area was disinfected using chlorhexidine digluconate gel 0.2% (Corsodyl, GSK B.V, Amersfoort, The Netherlands). A 1 mm biopsy punch was used to make a full-thickness wound located 1–2 mm posteriorly from the border of the nasopharyngeal sphincter close to the throat. Minor bleeding was removed using cotton swabs and saline water before implantation. 

**Figure 2:**
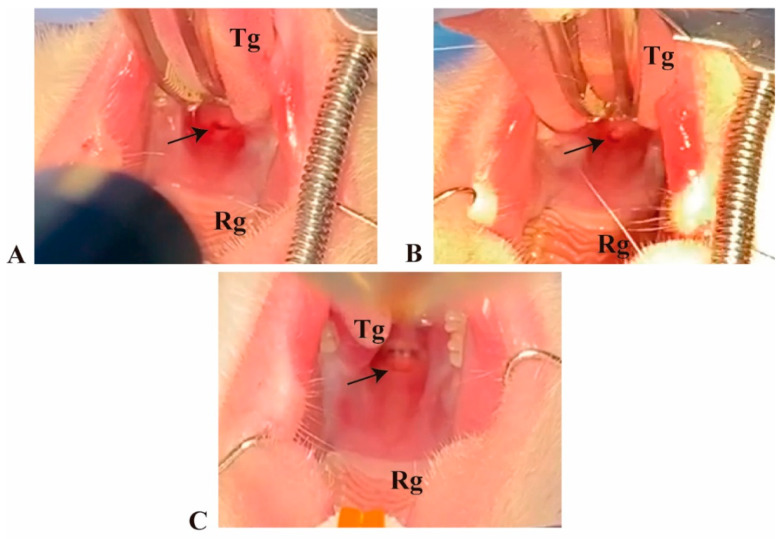
Fibrin construct at the day of wounding in the soft palate of the rat. The black arrows point to the wound site. (**A**) Excisional wound without implantation (W). (**B**) Fibrin-only implantation (WF). (**C**) Fibrin-laminin-nidogen implantation (WFL). Tg: tongue. Rg: Palatal rugae.

**Figure 3:**
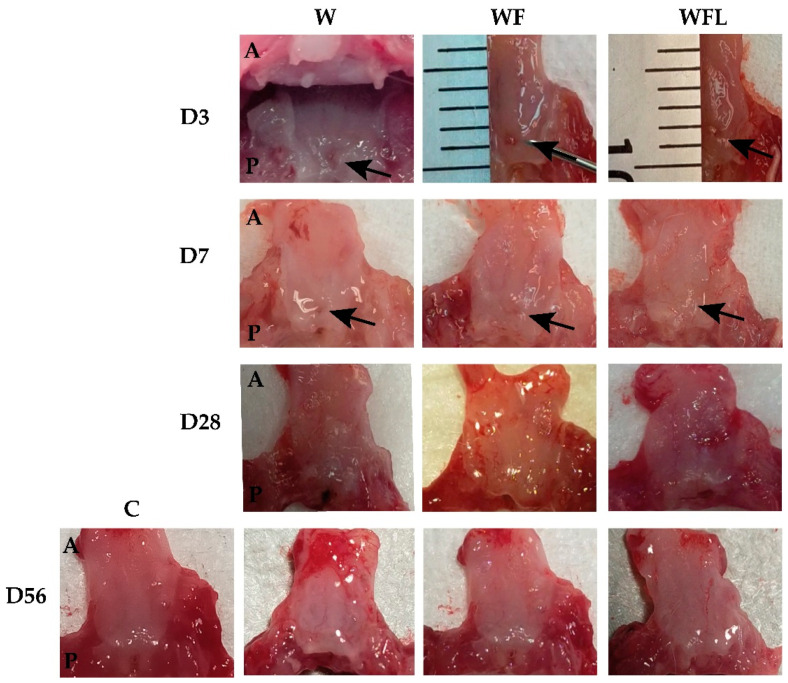
Representative images of the rat soft palate. Tissue samples from day 3 (D3), day 7 (D7), day 28 (D28), and day 56 (D56). A: anterior region of the soft palate. P: posterior side. Wound without implantation (W). Wounded soft palate with fibrin-only implantation (WF). Fibrin with laminin-entactin implantation (WFL). Age control (C) at day 56. D3-W shows the soft palate in the oral cavity during dissection. D3-WF and D3-WFL show the injury located approximately at 1 mm from the border of the soft palate. The arrows indicate the wound site in each sample. At days 7, 28 and 56 all the wounds already seem to be epithelialized.

The soft palate in rats is about 1mm thick including two outside mucosal layers with skeletal muscle and salivary glands in between [27]. A total of 4 μL of fibrin construct was slowly applied and left for 10 min allowing polymerization and attachment to the wound bed. No sutures were required since fibrin acts as a glue. Figure 2 and Figure 3 show fibrin implantation at day zero and subsequent timepoints.

Reversion of the anesthesia was obtained by atipamezole (Antisedan, Zoetis, Parsippany, NJ, USA) 1.25 mg/kg injected subcutaneously (0.1 mL/100 g). The animals were placed in a pre-warmed cage and monitored for respiratory arrest and postoperative bleeding. During the next three days after surgery, weight loss, dehydration and activity were followed up for each animal. Post-operative analgesia with carprofen (Rimadyl, Zoetis, Parsippany, NJ, USA) was provided in the drinking water. 

### 2.4. Immunohistology 

The soft palate of the rat was dissected as described previously [27]. In short, the levator veli palatini (LVP) muscle was detached from the lateral tympanic bullae. Then, the soft palate was detached from the 9th palatal rugae, the posterior border of the hard palate. After dissection, the samples were fixed in neutral buffered formalin 10% for 24 h and then transferred to ethanol 70% prior to automatic tissue processing (Leica TP1020, Leica Biosystems B.V., Amsterdam, the Netherlands) through a graded series of ethanol up to 100% followed by clearing in xylene, paraffin infiltration and paraffin embedding. After embedding, 300 sections of 5 µm thickness each were cut per palate. Every 25th serial section was stained with hematoxylin and eosin (H&E) to localize the wound (data not shown). Then, three sections per sample were placed on glass slides for each immunohistochemical and histochemical staining. 

Azocarmine G and aniline blue (AZAN) staining was used to differentiate connective tissue (blue) and muscle (red). Immunohistochemistry was used to detect myosin heavy chain (MyHC), alpha-smooth muscle actin (α-SMA) and paired box protein7 (Pax7) as described [27]. In summary, the sections were deparaffinized, dehydrated and immersed in H_2_O_2_ 3% in methanol for 20 min to destroy endogenous peroxidase. After rehydration, the sections were post-fixed in formalin 4% in PBS for 10 min. For MyHC antigen retrieval, the sections were immersed in TRIS/EDTA buffer pH 9.5, heated at 100 °C for 10 min and cooled down at room temperature. For α-SMA antigen retrieval, sections were immersed in TRIS/EDTA buffer pH 5.8, heated at 100 °C for 10 min and cooled to room temperature. For Pax7 antigen retrieval, citrate buffer pH 5.8 heated at 70 °C was used followed by trypsin treatment (0.075% in PBS at 37 °C for 5 min). The blocking was undertaken using normal donkey serum 10% in PBS plus glycine 0.075% (PBSG) for 30 min. Then, sections were incubated overnight at 4 °C in the primary antibodies (mouse anti-α-SMA (1:10,000, Chemical CO, St. Louis, MO, USA), mouse anti-fast MyHC (1:5000, Sigma Chemical CO, St. Louis, MO, USA), mouse anti-Pax7 (1:100, Developmental Studies Hybridoma Bank, Iowa City, CA, USA)). At the next day, the sections were rinsed with PBSG and incubated with a biotinylated secondary antibody donkey-anti-mouse IgG (H + L) (1:500; Jackson Labs West Grove, PA, USA). Finally, the sections were incubated in biotinylated horseradish peroxidase-avidin complex for 45 min (ABC-PO, Vector Laboratories, Burligame, CA, USA) and the detection was with 3,3-diaminobenzidine substrate for a maximum of 10 min. 

### 2.5. Digital Imaging, Image Analysis and Quantification

All stained sections were automatically scanned using a Pannoramic1000 scanner (3DHISTECH, Budapest, Hungary) with an AdimecQ camera (Adimec, Woburn, MA, USA) at standard settings. Digital images were visualized using CaseViewer-2.3 (3DHISTECH, Budapest, Hungary) and exported as TIFF files. For each image, the regions of interest (ROI) were determined as described [28]. In short, one central wound region of 700 µm was demarcated and the muscle layer was digitally isolated from the image. In the α-SMA staining, the salivary glands and the large blood vessels were excluded from the analysis. An Image J (Fiji, NIH-LOCI, Milwaukee, WI, USA) macro for each staining was applied as described [28,32,33]. The area measurements are expressed as the mean percentage ± SD of the ROI in mm^2^. 

### 2.6. Statistical Analysis

The stained areas in the different groups were compared by a two-way ANOVA and a Bonferroni post-hoc test. At day 56 the experimental groups were also compared with the age control group by a one-way ANOVA and a Bonferroni post-hoc test. The statistical analysis and the graphs were made in GraphPad Prism version 5 (GraphPad Software, San Diego, CA, USA; www.graphpad.com) (accessed on 24 August 2021). For all parameters, *p* values less than 0.05 were considered significant.

## 3. Results

### 3.1. Clinical Observations

The experimental animals showed a mild dehydration only at the first day after surgery (skin test). There were no prolonged bleedings and no signs of infection. In the two days after surgery the rats lost less than 15% of their body weight pre-surgery, which had recovered by day five. The body weight in the experimental rats at day 56 was not different from the age control.

### 3.2. Regenerative Effects of Fibrin Constructs Following Wounding 

**Figure 4:**
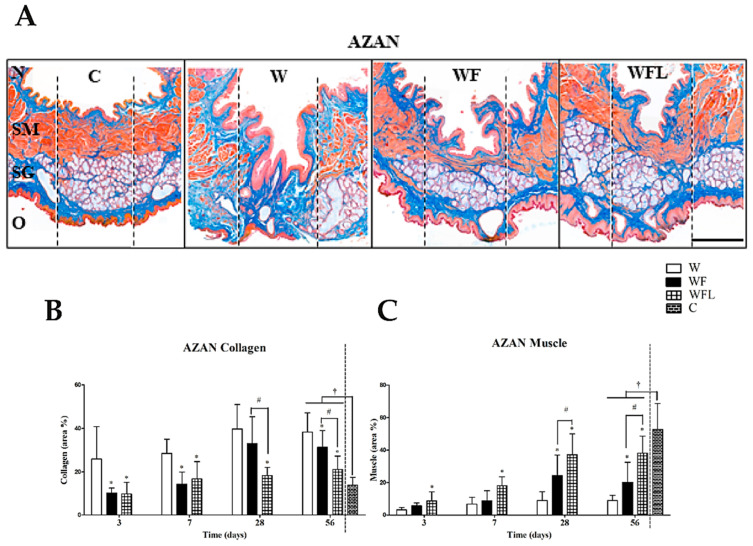
AZAN staining. (**A**) Representative images at day 56 (muscle in red, collagen in blue). Myofiber formation occurs mostly in fibrin-only (WF) and fibrin plus laminin-nidogen (WFL). N: nasal side of the soft palate. O: oral side of the soft palate. SM: skeletal muscle layer. SG: salivary glands. The vertical dotted lines indicate the central region of 700 µm. The lateral regions of 500 µm are next to it. Scale bar 500 µm. (**B**) Quantification of collagen. The area of collagen increased over time in all three experimental groups. At day 56 the collagen areas in all experimental wounds were higher than the age control, C (*p* ˂ 0.05). Data are shown as mean ± SD. (**C**) Quantification of muscle tissue. The area of muscle tissue in WFL increased more than in the other experimental groups but remained lower than the age control (*p* ˂ 0.05). The areas between the experimental groups were compared in time using a two-way ANOVA and a Bonferroni post-hoc test. Asterisks (*) indicate significant differences with the control wounds (W) (*p* ˂ 0.05). Hash (#) indicates significant differences between fibrin-only and fibrin plus laminin-nidogen (*p* ˂ 0.05). Comparisons between the experimental groups and the age control at day 56 (†) were made using a one-way ANOVA and a Bonferroni post-hoc test (*p*-value < 0.05).

The normal soft palate of the rat contains a pseudostratified respiratory epithelium at the nasal side and an epithelium that is keratinized and stratified at the oral side (Figure 4A,C). In between the mucosae, a muscle layer with the levator veli palatini (LVP) is located as well as a layer of salivary glands at the oral side. Inflammatory response was observed at day 3 that had decreased by day 7 in all experimental groups. There was no encapsulation of the fibrin constructs and no giant cells were observed (not shown). A complete re-epithelization of the oral and nasal mucosa in all experimental groups had occurred already after 3 days (not shown). The regenerated salivary glands often showed dilated ducts and absence of acini (Figure 4A SG). The salivary glands in the wounds treated with fibrin showed complete regeneration by day 56 (Figure 4A). In seven of the rats an epithelized oronasal communication or fistula had formed, W (n = 3), WF (n = 3), WFL (n = 1) that were excluded from further analysis. The images showed also a well-defined thick muscle layer in the age control (Figure 4A, SM) that is incomplete in the wounded soft palate (Figure 4A, W) and much further recovered in the fibrin implantation groups (Figure 4A, WF and WFL). All presented data below are derived from the central wound areas. 

The role of fibrin constructs on collagen deposition was measured as a marker of fibrosis. The collagen area was quantified in the samples stained with AZAN at days 3, 7, 28 and 56, and expressed as percentage of the total area (Figure 4B). The collagen area in the age control (C) was 13.89 ± 3.56%. At day 56, the collagen area in the control wounds (W, 38.25 ± 8.89%) was higher than in the wounds treated with fibrin-only (WF, 31.25 ± 7.73%, *p* ˂ 0.05) and the wounds with fibrin plus laminin-nidogen (WFL, 21.11 ± 6.06%, *p* ˂ 0.05). All these areas at day 56 were higher than the age control, *p* ˂ 0.05. The muscle area was measured at the same timepoints (Figure 4C). The muscle area in the age control (C) was 52.64 ± 15.94%. At day 56, the muscle area in W (9.05 ± 3.23%) was lower than in WF (20.14 ± 12.39%) and WFL (38.09 ± 10.59%) (*p* ˂ 0.05). All the muscle areas were lower than the age control, *p* ˂ 0.05.

### 3.3. Effects of Fibrin Constructs on Muscle Regeneration Following Wounding

**Figure 5:**
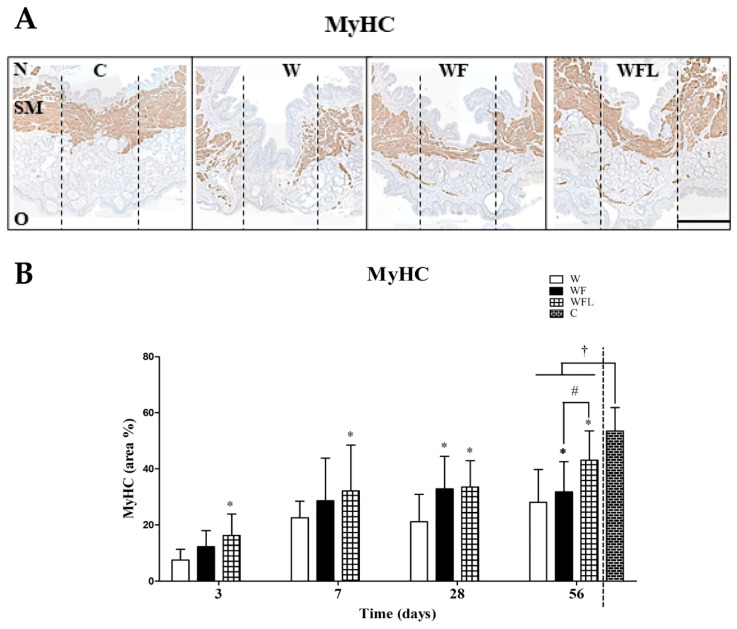
MyHC immunostaining. (**A**) Representative images at day 56. MyHC staining is shown in brown. MyHC expression is almost absent in the central region of the wounds without construct (W). N: nasal side of the soft palate. O: oral side of the soft palate. SM: skeletal muscle layer. The vertical black lines indicate the central region of 700 µm. The lateral regions of 500 µm are next to it. Scale bar: 500 µm. (**B**) Quantification of the MyHC staining. The MyHC area increases from day 3 to day 56 in all experimental groups. At day 56, the stained area in all experimental groups was lower than in the age control. Data are shown as mean ± SD. The areas between the experimental groups were compared in time using a two-way ANOVA and a Bonferroni post-hoc test. Asterisks (*) indicate differences with the control wounds (*p* ˂ 0.05). Hash (#) indicates differences between fibrin-only and fibrin plus laminin-nidogen (*p* ˂ 0.05). Comparisons between experimental groups and the age control at day 56 (†) were evaluated using a one-way ANOVA and a Bonferroni post-hoc test ( *p*-value < 0.05).

We also performed a MyHC immune staining to visualize specifically the layer of muscle fibers (brown) (Figure 5A). A clear muscle layer is observed in the age control group at day 56 (Figure 5A, C) that is almost absent in the control wounds (W) and partially recovered in the wounds with fibrin-only (WF) and fibrin plus laminin-nidogen (WFL). The area of newly formed myofibers in the wounds was quantified over time and expressed as a percentage of the total region in mm^2^ (Figure 5B). The area of the muscle layer in the age control is 53.44 ± 8.41%. At day 3 the muscle area in the WFL group was already higher than in the control wounds (W, *p* < 0.05). By days 7 and 28, the muscle areas in the wounds treated with fibrin-only (WF) and WFL increased over time, and both were higher than in the control wounds W (*p* ˂ 0.05). At day 56, the muscle area in the WF group (31.77 ± 10.81%) was lower than in the WFL group (43.13 ± 10.39%) but higher than in the control wounds W (28.10 ± 11.68%) (*p* ˂ 0.05). The muscle areas in all experimental groups were smaller than in the age control (C, *p* ˂ 0.05). 

### 3.4. Effects of Fibrin Constructs on α-SMA Expression Following Injury

**Figure 6:**
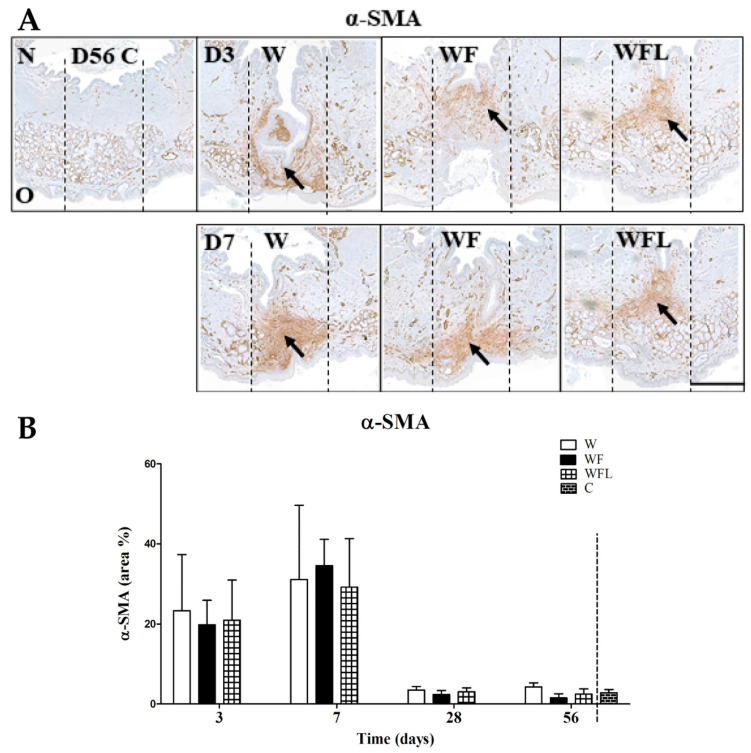
α-SMA immunostaining. (**A**) Representative images of the age control (C) at day 56 (D56) and the experimental groups at days 3 (D3) and 7 (D7). The age control (D56 C) at day 56 shows no staining in the central region except in small vessels and salivary glands. α-SMA expression by myofibroblasts is present mostly in the central wound regions and it seems to increase in all wounds from day 3 to 7. N: nasal side of the soft palate. O: oral side of the soft palate. The arrows indicate α-SMA expression in the wounds. The vertical black lines indicate the central region of 700 μm. The lateral regions of 500 μm are next to it. Scale bar is 500 µm. (**B**) Quantification of the staining. The stained areas increase from day 3 to 7 (*p* ˂ 0.05) without differences between the groups. By days 28 and 56 the stained areas in all experimental groups had decreased (*p* ˂ 0.05) without differences with the control wounds. Data are shown as mean ± SD. The areas between experimental groups were compared in time using a two-way ANOVA and a Bonferroni post-hoc test. Comparisons between experimental groups and the age control group at day 56 were performed using a one-way ANOVA and a Bonferroni post-hoc test.

The α-SMA (brown) staining detects myofibroblasts as well as vascular smooth muscle cells in blood vessels and myoepithelial cells of salivary glands but these latter two were excluded from analysis (Figure 6A). A high expression of α-SMA-positive myofibroblasts is observed at days 3 and 7 in all wounds (Figure 6A, arrows). At the later days, α-SMA expression was only observed in blood vessels and salivary glands (not shown). The area of α-SMA expression was measured and expressed as percentage of the total central region (Figure 6B). At day 3, the stained areas in all experimental groups were around 19% of the total wound region, while at day 7 the areas had increased to around 29% (*p* ˂ 0.05). At days 28 and 56 the stained areas had decreased to about 5% in all experimental wounds (*p* ˂ 0.05) without significant differences between the groups. 

### 3.5. Effects of Fibrin Constructs on Satellite Cell Recruitment Following Wounding

**Figure 7:**
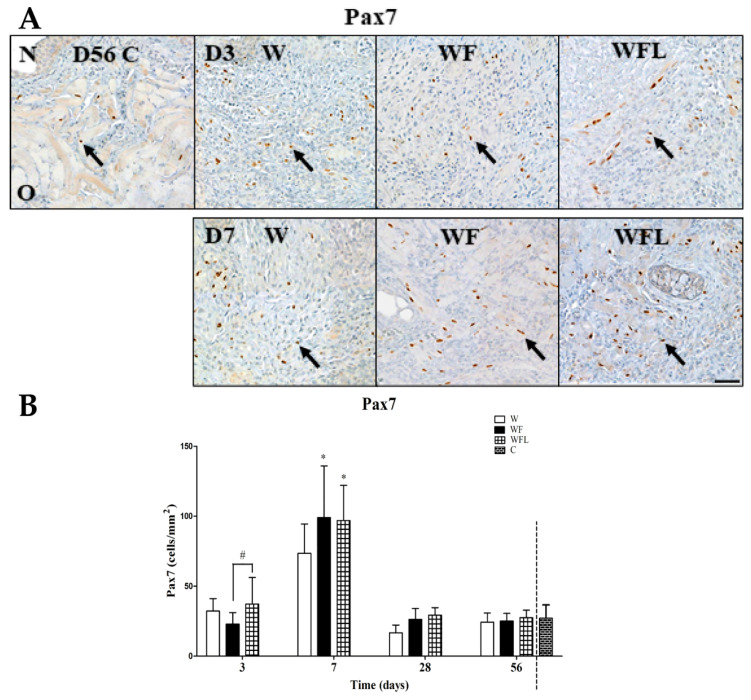
Pax7 immunostaining. (**A**) Representative images at days 56 (age control; D56), 3 (D3) and 7 (D7). Pax7 (brown, arrows) is mostly observed in the wounds treated with fibrin-only and fibrin plus laminin-nidogen at day 7. N: nasal side of the soft palate. O: oral side of the soft palate. Scale bar 50 µm. (**B**) Quantification of SCs. The Pax7 expression increases from day 3 to 7 in all experimental groups and had decreased by days 28 and 56 (*p* ˂ 0.05). At day 56 there were no differences with the age control. Data are shown as mean ± SD. The experimental groups were compared in time using a two-way ANOVA and a Bonferroni post-hoc test. Asterisks (*) indicate significant differences with the control wounds (*p* ˂ 0.05). Hash (#) indicates significant differences between fibrin-only and fibrin plus laminin-nidogen (*p* ˂ 0.05). Comparisons between experimental groups and the age control at day 56 were made using a one-way ANOVA and a Bonferroni post-hoc test.

The paired homeobox protein 7 (Pax7) is a marker of SCs and is shown in brown (Figure 7A). In the age control few positive cells are observed close to large muscle fibers. Pax7-positive cells are mostly observed at day 7 in the wounds treated with fibrin-only (WF) and fibrin with laminin-nidogen (WFL), mainly near small myofibers. The number of Pax7-positive cells were counted at days 3, 7, 28 and 56 (Figure 7B). The number of positive cells in the age control (C) is 27.13 ± 9.53. At day 3, the number of positive cells is already higher in the wounds treated with fibrin plus laminin-nidogen (WFL) than in the fibrin-only group (WF) (Figure 7B, arrows) (*p* ˂ 0.05). At day 7, the number of Pax7-positive SCs in the WF group (99.0 ± 36.87) and WFL group (96.90 ± 25.13) was higher than in the control wounds (W) (73.33 ± 21.04) (*p* ˂ 0.05). At days 28 and 56, the number of positive cells in the experimental groups was not different from each other and from the age control (C) (Figure 7B). 

## 4. Discussion

This study aimed to evaluate the effects of fibrin constructs with or without laminin-nidogen on fibrosis and myofiber regeneration in the wounded soft palate. As a measure for fibrosis, we analyzed collagen deposition as well as the presence of myofibroblasts. To evaluate muscle regeneration, we analyzed muscle quantity and the numbers of Pax7-positive satellite cells. Generally, our findings show that the implantation of fibrin enhanced with laminin-nidogen improves the regeneration of the soft palate by limiting fibrosis and stimulating myofiber formation. 

Fibrin has been applied to wounds in different forms such as micro and nanoparticles, nanofibers, microtubes, sealants and hydrogels [34]. The electromechanical stimulation of fibrin scaffolds seems to further improve the regeneration of limb muscle defects [35]. In clinical practice, fibrin is widely applied as a sealant or glue during surgery. Platelet-rich fibrin is also used in maxillofacial surgery, mostly for bone regeneration [36]. Additionally, it is used to treat sports injuries without muscle loss [36].

Our findings show that, in the control wounds, the muscle layer was mainly replaced by collagenous tissue indicating fibrosis. This confirms our earlier findings in this rat model [28]. Several other animal studies in skeletal muscles from limb and trunk also show that excisional wounds cause fibrosis [20,33,37]. These studies also show reduced myofiber formation and a high number of myofibroblasts at one week post-wounding. In general, fibrosis after tissue injury is induced by transforming growth factor-β1 (TGF-β1) that stimulates fibroblasts to differentiate into myofibroblasts, contract the tissue and produce large amounts of collagen [38,39]. Additionally, in muscle injuries, TGF-β1 fuels the fibrotic process [3,40,41]. Excisional wounding induces extensive fibrosis in skeletal muscles including the soft palate, while muscle regeneration is impaired [28]. 

In our study, fibrin alone as well as fibrin with laminin-nidogen show reduced collagen deposition in the wounds. This effect is remarkable as fibrin is a main component of the normal blood clot that forms after injury [42]. In an untreated wound, the fibrin clot is degraded within a few days and replaced by granulation tissue [38]. Therefore, the implanted fibrin alone already seems to influence early events in fibrosis. This may be due to the lack of other components of the normal blood clot such as platelets. In vitro, platelet-derived polyphosphates induce the differentiation of fibroblasts from skin into myofibroblasts [43]. Moreover, platelets contain large amounts of TGF-β1, a key factor in fibrosis and scarring. [44,45]. Therefore, the lack of platelets in the implanted fibrin might explain the reduced fibrosis. An additional antifibrotic effect in our experiments seems to be induced by the addition of laminin-nidogen. In muscular dystrophies, such as the laminin myopathies, fibrosis is caused by a mutated laminin that impairs the basement membrane structure leading to extensive inflammation [46]. These data and ours indicate that a functional laminin reduces the fibrotic process by allowing correct formation of the basement membrane and thus reducing inflammation. 

Similarly, in a nidogen-1 deficient mouse model, alteration of the laminin distribution, impairment of the basement membrane structure, and an increase of α-smooth muscle actin levels was found [47]. Nidogen binds laminins and collagen-IV to stabilize the basement membrane [48]. In vitro, nidogen stimulates the expression and deposition of laminin and its binding to collagen-IV in cultures of epithelial cells [48,49]. Thus, both laminin and nidogen seem to be critical for the assembly and maintenance of the basement membrane. Disruption of the basement membrane leads to inflammation and subsequently fibrosis. Therefore, the addition of laminin-nidogen to our constructs might explain the reduced fibrotic process. In short, the reduced fibrosis in our model seems to be related to the absence of platelets and the antifibrotic effects of laminin and nidogen. 

The reduction of fibrosis by laminin-nidogen alone might already allow more efficient myofiber formation. However, also direct specific effects of laminin-nidogen on myofiber formation may also contribute. In vitro binding assays showed that the binding of laminin to myostatin inhibits its activity, which increases myofiber formation [50]. Myostatin is part of the TGF-β superfamily and inhibits myofiber formation, whereas its inactivation promotes muscle regeneration [50,51]. In vivo, *mdx* mice that also have a mutation in myostatin show increased myofiber formation similar to wildtype mice [52]. In immunodeficient mice with a limb muscle injury induced by irradiation, the injection of myoblasts in combination with laminin-1 also enhanced myofiber formation [53]. Moreover, fibrin with laminin-111 implanted in an excisional wound in a mouse limb muscle increased myofiber formation [24]. All this suggests that the inhibition of myostatin by laminin may have contributed to the stimulated myofiber formation in our palatal regeneration model. 

Additionally, nidogen may stimulate myofiber formation as observed in studies using C2C12 myoblasts [54,55]. In vitro, satellite cells and myoblasts bind nidogen and other basement membrane components, which enhances their adhesion to laminin and promotes their proliferation and early differentiation [49,56,57,58,59]. Other studies have shown that nidogen also stimulates laminin expression in vitro by myoblasts and fibroblasts [48,55]. In our in vivo study, nidogen may also facilitate myoblast adhesion to laminin early after wounding as well as later myofiber formation. In summary, our fibrin constructs with laminin and nidogen seem to reduce fibrosis by direct effects of fibrin that are now visible because of the absence of platelets and stimulation of basement membrane assembly by laminin-nidogen. Myofiber formation may be further promoted by laminin-nidogen through inhibition of myostatin and stimulation of myoblast adhesion and early differentiation. 

The function of the soft palate decreases after surgical palatal injury as shown by electromyography (EMG) in rodents and humans [29,60]. This might also be related to the reduced capacity for regeneration of head muscles as compared to other skeletal muscles [3]. In addition, head muscles tend to develop more fibrosis after injury. Therefore, specific strategies to improve the regeneration of head muscles after reconstructive surgery are required. Generally, strategies to improve skeletal muscle regeneration involve the use of biomaterials to deliver therapeutic molecules, antifibrotic compounds, and stem cells [40,61].

An additional finding from the current study was that the salivary glands in the wounds treated with the fibrin constructs regenerated faster than in the control wounds. In the control wounds, the acini seemed to be smaller or absent and the ducts enlarged. In contrast, the fibrin constructs-treated wounds showed a complete layer of salivary glands. The regeneration of salivary glands mainly depends on multipotent epithelial progenitor cells that differentiate into α-SMA-positive myoepithelial cells, and the cells that form the acini and the ducts [62]. Acini and ducts are supported by a basement membrane containing laminin, nidogen and collagen-IV [63]. In a recent in vivo study, laminin-111 promoted the regeneration of the submandibular gland in mice after excisional wounding [64]. The effect of laminin might thus be related to a faster assembly of the basement membrane supporting the increased regeneration of salivary glands.

## 5. Conclusion

The implantation of fibrin constructs with laminin-nidogen reduced fibrosis and stimulated muscle and salivary gland regeneration in our in vivo model for palatal fibrosis and regeneration. The use of enhanced acellular fibrin constructs might be more efficient and cost-effective than constructs with isolated cells. This seems to be a promising strategy to improve soft palate regeneration after cleft palate surgery. Similar strategies might be employed to improve the regeneration of other severely injured skeletal muscles.

## Data Availability

All relevant data supporting the findings of this study are available within the paper. The data that support the findings of this study are available from the corresponding author upon reasonable request. All raw and processed data digitally recorded on the local server, within the department of Dentistry, Radboud University Medical Center, Nijmegen (NL), THK_RE5$(umcms36) folder 2016024/private.
